# Predicting Neural Response Latency of the Human Early Visual Cortex from MRI-Based Tissue Measurements of the Optic Radiation

**DOI:** 10.1523/ENEURO.0545-19.2020

**Published:** 2020-07-02

**Authors:** Hiromasa Takemura, Kenichi Yuasa, Kaoru Amano

**Affiliations:** 1 Center for Information and Neural Networks (CiNet), National Institute of Information and Communications Technology, and Osaka University, Suita-shi, Osaka 565-0871, Japan; 2 Graduate School of Frontier Biosciences, Osaka University, Suita-shi, Osaka 565-0871, Japan; 3 Department of Psychology, New York University, New York, NY 10003

**Keywords:** diffusion MRI, optic radiation, primary visual cortex, quantitative T1, visually evoked response

## Abstract

Although the non-invasive measurement of visually evoked responses has been extensively studied, the structural basis of variabilities in latency in healthy humans is not well understood. We investigated how tissue properties of optic radiation could predict interindividual variability in the latency of the initial visually evoked component (C1), which may originate from the primary visual cortex (V1). We collected C1 peak latency data using magnetoencephalography (MEG) and checkerboard stimuli, and multiple structural magnetic resonance imaging (MRI) data from 20 healthy subjects. While we varied the contrast and position of the stimuli, the C1 measurement was most reliable when high-contrast stimuli were presented to the lower visual field (LVF). We then attempted to predict interindividual variability in C1 peak latency in this stimulus condition with a multiple regression model using MRI parameters along the optic radiation. We found that this model could predict >20% of variance in C1 latency, when the data were averaged across the hemispheres. The model using the corticospinal tract did not predict variability in C1 latency, suggesting that there is no evidence for generalization to a non-visual tract. In conclusion, our results suggest that the variability in neural latencies in the early visual cortex in healthy subjects can be partly explained by tissue properties along the optic radiation. We discuss the challenges of predicting neural latency using current structural neuroimaging methods and other factors that may explain interindividual variance in neural latency.

## Significance Statement

Although the temporal properties of visually evoked responses has been studied extensively, the structural basis of variabilities in latency measured in healthy humans is not well understood. Here we tested how the properties of the optic radiation could predict interindividual variability in the latency of the initial visually evoked component (C1). We found that magnetic resonance imaging (MRI) measurements on the optic radiation could partly predict interindividual variability in C1 latency, while MRI measurements on the corticospinal tract did not. Overall, our work demonstrates that variability of neural latency in the early visual cortex of healthy humans can be partly explained by neuroimaging measurements of tissue properties along the optic radiation, although there are remaining challenges to explain latency variabilities from structural neuroimaging.

## Introduction

Non-invasive measurement methods, such as electroencephalography (EEG) and magnetoencephalography (MEG), have been widely used to quantify the temporal properties of human cortical responses (Norcia et al., 2015; Hämäläinen et al., 1993; Baillet, 2017). One of the traditional approaches has been visually evoked responses, which measure the neural response evoked by visual stimuli using EEG or MEG (Adrian and Matthews, 1934). This approach has been confirmed to be useful for understanding the neural dynamics underlying visual perception (Amano et al., 2006), attentional modulation of visual processing (Hillyard and Anllo-Vento, 1998), and the development and impairment of the visual system (Braddick et al., 1986; Anderson et al., 1999). Studies using this approach have revealed several major components in human visually evoked responses with a specific range of peak latencies. The variability in these components’ latencies provides essential information to understanding the functional properties and disorders of the visual system (Halliday et al., 1972; Thurtell et al., 2009). Measurements of these components evoked by visual stimuli presented to a specific visual field have also been used to assess visual field loss (Klistorner et al., 1998). Among major components, the earliest is the C1 (also known as N75), which appears in channels located near the occipital pole with a peak latency of 60–100 ms following stimulus onset (Clark et al., 1994; Di Russo et al., 2002). Numerous studies have reported that the cortical source of C1 is the primary visual cortex (V1; Jeffreys and Axford, 1972; Clark et al., 1994; Di Russo et al., 2002). However, while the temporal properties and cortical origins of major visually evoked components have been extensively studied, the question of why healthy humans show large interindividual differences in peak latency of visually evoked responses, even in the earliest component, remains unanswered.

Neurobiological studies suggested that the signal transmission efficiency (conduction velocity) along a long-range axon depends on the microstructural properties of white matter, such as the morphologic properties of the myelin sheaths or axons (Pumphrey and Young, 1938; Cullheim and Ulfhake, 1979; Waxman, 1980; Etxeberria et al., 2016). We hypothesized that the interindividual difference in latency of the visually evoked response may be at least partly explained by differences in the tissue in the white matter tracts, which carry signals to visual areas in the cortex.

Recent advances in non-invasive structural magnetic resonance imaging (MRI) and tractography have in part enabled us to measure the tissue properties of white matter and the trajectory of the major white matter pathways. Computational modeling of diffusion-weighted MRI (dMRI) signals provides a variety of structural measurements, ranging from those using a simpler diffusion tensor model (Basser and Pierpaoli, 1996) to those using advanced multicompartment models (Zhang et al., 2012). The recent advent of quantitative T1 (qT1) mapping methods also provides quantitative metrics on white matter tissue properties (Mezer et al., 2013; Weiskopf et al., 2015). However, it is not fully understood how these MRI-based structural measurements along the visual pathway are related to the interindividual variability of C1 latency in healthy subjects.

The visual system is an excellent model system to test a hypothesis concerning microstructural measurements in white matter and functional measurements of neural latencies because both the anatomy of the white matter tract and the major evoked response components are relatively well understood. Moreover, recent advances in tractography algorithms have improved sensitivity for identifying the optic radiation, which carries signals from the lateral geniculate nucleus (LGN) to the V1, from a dMRI dataset (Sherbondy et al., 2008b; Chamberland et al., 2017). The optic radiation has a larger volume and a relatively higher signal-to-noise ratio and is less affected by susceptibility-induced distortions in dMRI measurements than other fiber tracts, i.e., the optic nerve and optic tract. Therefore, we assumed that the optic radiation would be a suitable model pathway to test how MRI-based tissue measurements from the white matter tracts could explain the variability in human V1 response latency, which can be measured as C1 latency using MEG.

To this end, we collected visually evoked response data using MEG and structural data for the optic radiation using dMRI and qT1 from 20 healthy subjects. We analyzed how measurements in the optic radiation may predict the interindividual variability of C1 latencies. The goal of this study was to test the extent to which MRI-based tissue measurements along the optic radiation could predict interindividual variability in C1 peak latency to understand the extent to which non-invasive structural measurements can explain variability in neural latency in the early visual cortex.

## Materials and Methods

### Subjects

Twenty healthy volunteers (15 males, 5 females; age mean ± SD, 28.6 ± 7.96 years old; ranging from 21 to 53 years old) participated in the study. All subjects had a normal or corrected-to-normal vision. dMRI, qT1, and MEG data were collected on different days. The study protocol was approved by the local ethics and safety committees at Center for Information and Neural Networks (CiNet), National Institute of Information and Communications Technology (NICT), and conducted in accordance with the ethical standards stated in the Declaration of Helsinki. Written informed consent was obtained from all study subjects.

### MRI experiments

We collected T1-weighted MRI, dMRI and qT1 data from all subjects. We note that many parts of MRI acquisition method is common to those used in our previous works (Oishi et al., 2018; Minami et al., 2020).

#### T1-weighted MRI data acquisition and tissue segmentation

We obtained T1-weighted magnetization-prepared rapid gradient-echo (MP-RAGE) images (1 mm isotropic; repetition time, 1900 ms; echo time, 2.48 ms) from all subjects using a 3T SIEMENS Prisma/Trio scanner at CiNet, NICT to estimate the border between white matter and gray matter. The acquisition of T1-weighted MRI data took around 15 min for each subject. The tissue segmentation was performed using an automated procedure implemented in FreeSurfer software (https://surfer.nmr.mgh.harvard.edu/; Fischl, 2012). The tissue segmentation was used for subsequent dMRI and MEG analysis.

#### Diffusion MRI data acquisition

We measured dMRI data from all subjects. All dMRI data were acquired using the 3T Magnetom Prisma scanner (Siemens) with a 32-channel head coil at CiNet, NICT. The dMRI data were acquired using monopolar single-shot echo-planar imaging (EPI; repetition time, 3300 ms; echo time, 66.4 ms; multiband factor, 3; partial Fourier, 5/8; voxel size, 2 × 2 × 2 mm^3^) implemented in a multiband accelerated EPI pulse sequence provided by the Center for Magnetic Resonance Research, Department of Radiology, University of Minnesota (Setsompop et al., 2012; https://www.cmrr.umn.edu/multiband/). The diffusion weighting was isotropically distributed along six directions at *b *=* *300 s/mm^2^, 30 directions at *b *=* *1000 s/mm^2^, and 64 directions at *b *=* *2000 s/mm^2^. Eight non-diffusion-weighted (*b *=* *0) images were acquired per image set. To minimize EPI distortion, two image sets were acquired with reversed phase-encoding directions (anterior-posterior and posterior-anterior). The entire dMRI data acquisition took around 25 min for each subject.

#### qT1 data acquisition

We measured qT1 data for all subjects. The qT1 data were acquired using the 3T Magnetom Trio scanner (Siemens) with a 32-channel head coil at CiNet, NICT and the protocols described in previous publications (Mezer et al., 2013; Gomez et al., 2017; Bain et al., 2019; Filo et al., 2019). We acquired four fast low-angle shot (FLASH) images with flip angles of 4°, 10°, 20°, and 30° (repetition time, 12 ms; echo time, 2.41 ms) and an isotropic scan resolution of 1 mm. For the purposes of removing field inhomogeneities, we further collected five additional spin echo inversion recovery (SEIR) images with an EPI readout (repetition time, 3 s; echo time, 49 ms; 2× acceleration) with the inversion times of 50, 200, 400, 1200, and 2400 ms. The in-plane resolution and the slice thickness of the additional SEIR scan were 2 × 2 mm^2^ and 4 mm, respectively. The entire qT1 acquisitions took around 35 min for each subject.

#### MEG experiment

##### MEG data acquisition

In a magnetically shielded room, we measured visually evoked responses from all subjects using a 360-channel whole-head MEG system (Neuromag 360, Elekta) at CiNet, NICT. The MEG system consists of 204 planar gradiometers, 102 magnetometers, and 54 additional sensors for noise reduction. Magnetic signals were recorded at a sampling frequency of 1000 Hz. Both planar gradiometers and magnetometers were used for the analysis.

#### Visual stimuli and task design

##### Apparatus

Visual stimuli were presented using an LCD projector (PT-DZ680, Panasonic) on a translucent screen in a magnetically shielded room. Gamma correction on the LCD projector was performed using Mcalibrator2 software (Ban and Yamamoto, 2013; https://github.com/hiroshiban/Mcalibrator2). The projector spanned 27.6 × 20.7° of the visual angle (1024 × 768 resolution) and had a 60-Hz refresh rate. The viewing distance was 61 cm. Subjects who used glasses wore plastic correction lenses during all MEG measurements. All stimuli were generated using the MATLAB programming environment (MathWorks) and the Psychophysics Toolbox 3 routines (https://github.com/Psychtoolbox-3/Psychtoolbox-3; Brainard, 1997).

##### Visual stimuli

**Figure 1.**
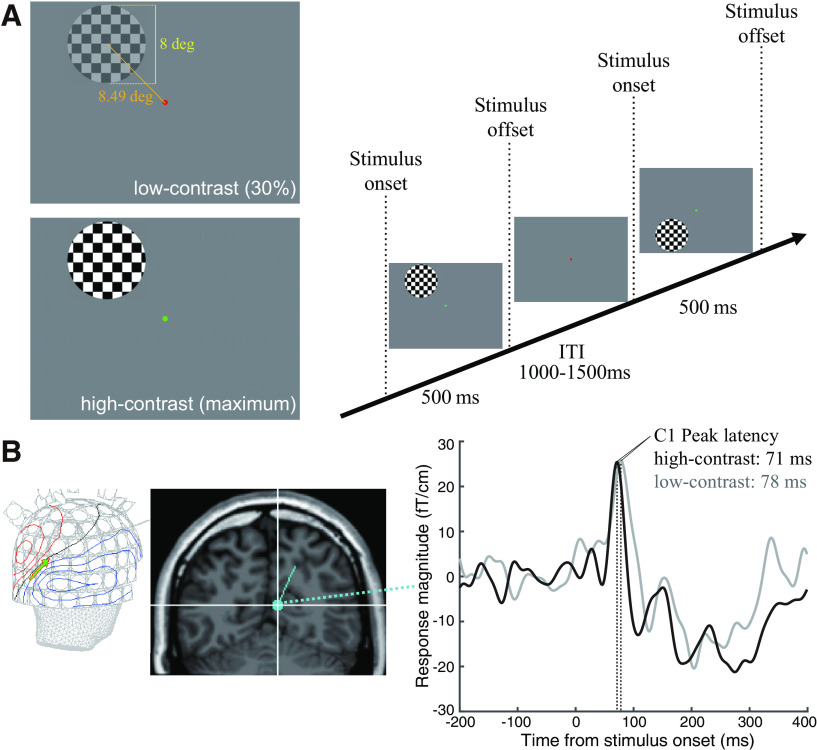
MEG experiment and data analysis. ***A***, Visual stimuli used in the MEG experiment measuring the visually evoked response. Left panel, Example of a checkerboard visual stimulus with low-contrast (upper panel, 30% contrast) and high-contrast (lower panel, maximum contrast). Right panel, The sequence of trials. In each trial, the checkerboard stimuli were presented for 500 ms with an inter-trial interval (ITI) of 1000–1500 ms. During the experiment, subjects were asked to maintain fixation at the red or green dot in the center of the screen and perform a fixation task. ***B***, Left panel, The contour map of magnetic fields in a representative subject (subject 2, left LVF stimulation, high contrast; 71 ms after the stimulus onset). The blue and red contours represent the sink and source of the magnetic fields, respectively. The green arrow represents the location and direction of the ECDs projected on the skull surface. The contour map represents the dipolar field pattern near the occipital pole, suggesting a single source of cortical activity, rather than distributed activity. Middle panel, Example of a C1 dipole (left panel) overlaid on a coronal slice of a T1-weighted image (subject 2, left LVF stimulation, high-contrast). The location of the C1 dipole (blue dot) is near V1 (calcarine sulcus) in the contralateral hemisphere. Right panel, The response time course of the C1 dipole (subject 2, left LVF stimulation; black curve, high-contrast; gray curve, low-contrast). We note that the location of C1 dipole in low-contrast condition was similar but not identical to that shown in the middle panel, since C1 dipole was separately estimated in each condition (see Materials and Methods). In each condition, the C1 peak latency was determined as the time period when the response amplitude of an ECD reached the maximum.

The stimuli (Fig. 1*A*) consisted of square-wave circular checkerboards; each stimulus had a diameter of 8° of visual angle and spatial frequency modulation of 0.5 cycles per degree. Eccentricity at the center of the stimuli was 8.49°. The subjects were asked to maintain fixation on the fixation point (diameter, 0.2°) presented at the center of the screen. Stimulus positions were centered along an arc that was equidistant (8.49°) from a central fixation point and located at polar angles of 45° above or below the horizontal meridian (Fig. 1*A*). The stimuli were presented at one of four positions (upper left, lower left, upper right, lower right) and at low or high luminance contrast values (30% or maximum Michelson contrast). Visual stimuli were presented binocularly at one of four quadrants and with one of the stimulus contrasts in a randomized sequence. The stimulus duration was 500 ms. The inter-trial interval was varied between 1000 and 1500 ms. Each session consisted of 144 trials (18 trials for each stimulus condition).

The total length of each session was ∼250 s. All subjects were tested with 12 such sessions, resulting in 216 trials for each stimulus condition. The subjects were able to take a break between sessions whenever needed. During the MEG experiment, we logged the stimulus onset of each trial by using a photodiode, which was used to precisely align the MEG signals to the timing of the onset for each stimulus. The entire MEG acquisitions took around 67 min for each subject.

##### Task

To maintain an adequate level of alertness and a stable fixation during the experiment, each subject was asked to perform a fixation task. The subject was instructed to press a button when the color of the fixation point (green or red) changed. The change in the fixation color occurs randomly during each session, with no systematic relationship to stimulus onset or offset. On average, the fixation color changed 61.9 times during each session. The subjects were able to respond to a change in the fixation color within 1 s on 86.4% of occasions on average.

### Data analyses

We note that many parts of MRI data analysis pipeline are common to those used in our previous works (Oishi et al., 2018; Takemura et al., 2019; Minami et al., 2020).

#### Diffusion MRI data analysis

##### Preprocessing

dMRI images were corrected for susceptibility-induced distortions using FSL TOPUP (Andersson et al., 2003) based on non-diffusion-weighted images acquired with reversed phase-encoding directions. dMRI images were further corrected for eddy-current distortions and subject motion using FSL EDDY (Andersson and Sotiropoulos, 2016).

##### Quantification of tissue measurements

We fitted the diffusion tensor model to the dMRI data using iteratively reweighted linear least squares estimation (Veraart et al., 2013) implemented in MRTrix3 (http://www.mrtrix.org/; Tournier et al., 2012, 2019). We computed the eigenvalue decomposition of the diffusion tensor imaging (DTI; Basser and Pierpaoli, 1996), and the resulting eigenvalues were used to compute the fractional anisotropy (FA) and mean diffusivity (MD). Furthermore, we fitted a multicompartment model, neurite orientation dispersion and density imaging (NODDI; Zhang et al., 2012), to the dMRI data using the NODDI MATLAB toolbox (http://mig.cs.ucl.ac.uk/index.php?n=Tutorial.NODDImatlab) to obtain orientation dispersion index (ODI) and intracellular volume fraction (ICVF) maps.

##### Tractography on the optic radiation

We identified the optic radiation using a dedicated method (ConTrack; Sherbondy et al., 2008a) in view of the known challenges when estimating the human optic radiation using whole-brain tractography, particularly tracking in crossing fiber regions at around the Meyer’s loop (Chamberland et al., 2017). First, we estimated the approximate location of the LGN by manual inspection of a T1-weighted MRI data and deterministic tractography from the optic chiasm (Ogawa et al., 2014; Takemura et al., 2019). We then placed an 8-mm radius sphere that covered the whole LGN endpoints of streamlines from the optic chiasm. Second, we identified the location of the V1 using a probabilistic atlas of retinotopic visual areas (Wang et al., 2015). Specifically, we performed surface-based registration between atlas surface and individual subjects' surface created by tissue segmentation on T1-weighted MRI data, in order to identify the V1 in individual subjects. Using ConTrack, we then sampled 100,000 candidate streamlines connecting LGN and V1 (angle threshold, 90°; step size, 1 mm). Tracking was restricted based on the white matter mask generated by tissue segmentation. We selected the top 30,000 streamlines with higher scores in the ConTrack scoring process (Sherbondy et al., 2008b). We further excluded streamlines that had either (1) a streamline length of ≥5 SD longer than the mean streamline length in the tract, or (2) a streamline position of ≥5 SD away from the mean position of the tract (Yeatman et al., 2012). We identified the optic radiation of each subject separately for two dMRI sessions with reversed phase encoding directions. We then merged the streamlines identified from the two dMRI sessions (Oishi et al., 2018). Further details on the methods used to identify the optic radiation with ConTrack are described in previous papers (Sherbondy et al., 2008b; Levin et al., 2010; Duan et al., 2015; Malania et al., 2017; Takemura et al., 2019).

##### Tractography on the corticospinal tract

The corticospinal tract was used as a control in the analysis since it has no terminations in the occipital cortex, is large in volume, and has a relatively higher signal-to-noise ratio. We used multi-shell multi-tissue constrained spherical deconvolution (*L_max_* = 8; Jeurissen et al., 2014) on the dMRI data to estimate fiber orientation distribution in each voxel using MRTrix3 (Tournier et al., 2012, 2019). We performed probabilistic tractography implemented in MRTrix3 to generate 2 million candidate streamlines for each dMRI image set (step size, 1 mm; maximum angle between successive steps, 45°; minimum length, 10 mm; maximum length, 250 mm; fiber orientation distribution amplitude stopping criterion, 0.05). The seed voxels for tracking were randomly chosen from the whole-brain white matter mask created by tissue segmentation on T1-weighted MRI data. We identified the corticospinal tract from whole-brain streamlines using automated procedure implemented in the AFQ toolbox (https://github.com/yeatmanlab/AFQ; Yeatman et al., 2012) including the outlier streamline exclusion process. Specifically, after identifying the corticospinal tract, we further excluded streamlines that had either (1) a streamline length of ≥3 SD longer than the mean streamline length in the tract, or (2) a streamline position of ≥3 SD away from the mean position of the tract. We used relatively conservative exclusion criteria for the corticospinal tract since, unlike optic radiation streamlines, streamlines did not pass through another exclusion step (the ConTrack scoring). We identified the corticospinal tract of each subject separately for two dMRI sessions with reversed phase encoding directions. We then merged the streamlines identified from the two dMRI sessions.

#### qT1 data analysis

The FLASH and SEIR images were processed using the mrQ software package (https://github.com/mezera/mrQ) in MATLAB to compute the qT1 map. In brief, qT1 maps were calculated based on variable flip angles which were corrected for B1 excite inhomogeneity based on the unbiased SEIR data (Barral et al., 2010). A description of the full analysis pipeline on qT1 data analysis can be found in a previous publication (Mezer et al., 2013).

#### MEG data analysis

##### Preprocessing

The recorded MEG signals were first spatiotemporally filtered with the temporal signal space separation (tSSS) method (Taulu et al., 2005; Taulu and Hari, 2009) using Maxfilter 2.2.15 (Elekta Neuromag Oy) after removal of the bad channels detected by MEG Xscan tools (Elekta Neuromag Oy). Using Maxfilter, we also roughly transformed the head position in individual subject’s data to the head position of a representative subject (subject 1). This process ensures a common channel selection procedure across all subjects in subsequent steps (see “Equivalent current dipole (ECD) estimation and identification of C1 peak latency” below). We further applied bandpass filtering (from 1 to 40 Hz) to MEG signals, which were then averaged across 216 trials under each stimulus condition.

##### Equivalent current dipole (ECD) estimation and identification of C1 peak latency

We used the single-ECD model to estimate the cortical origin of C1 from MEG signals, using the xfit tool (Elekta Neuromag Oy). We chose the single-ECD model because it is an adequate method for the localization of the magnetic field generated by a single localized source, such as in the context of early sensory or motor evoked responses (Salmelin and Hari, 1994; Di Russo et al., 2002; Parkkonen et al., 2009; Maezawa et al., 2016), as compared with methods better suited to distributed cortical responses (e.g., minimum norm estimates methods; Hämäläinen and Ilmoniemi, 1994). We selected 60 channels at 20 locations (a magnetometer and two gradiometers at each location) for performing the single-ECD analysis. We selected sensors that showed larger visually evoked activation in each stimulus condition based on averaged data for all 20 subjects. After selecting identical pairs of channels across all subjects, an ECD was estimated for single subject data. We performed single dipole fitting to each stimulus condition’s data (with regard to stimulus position and contrast) sequentially from 50 to 100 ms following stimulus onset. This latency range was chosen for adequate coverage of the onset and peak of C1 reported in a previous study (Di Russo et al., 2002). The dipole fitting was performed with a boundary element method volume conductance model of the individual subject’s head based on T1-weighted MRI data. We then selected the ECD with the best goodness-of-fit (GOF) as the representative ECD of C1. In most cases, a single dipolar magnetic field pattern was found in the occipital cortex at around the GOF peak (Fig. 1*B*, left panel), and the ECD was estimated along the calcarine sulcus (Fig. 1*B*, middle panel). We then fixed the dipole position and orientation of the representative ECD over the entire time interval, and estimated the time course of the ECD amplitude (Fig. 1*B*, right panel). We identified C1 peak latency as the time period when the ECD amplitude reached maximum (see Fig. 1*B*). C1 peak latency was estimated separately for each stimulus condition [left/right upper visual field (UVF) or lower visual field (LVF); low- or high-contrast]. In the main analyses, C1 latency was further averaged across the left and right visual field presentations.

##### Assessment of test-retest reliability

We assessed C1 latency measurement’s test-retest reliability by separating MEG data in each stimulus condition into odd and even trials (108 trials for each). We estimated the C1 dipole separately for odd and even trials using a single-ECD model and identified the C1 peak latency. The C1 latencies identified for left and right visual field presentations were averaged. Finally, we evaluated the degree of test-retest reliability by measuring the interindividual correlation (*R*) of C1 peak latency between the odd and even trials in four stimulus conditions (UVF/low-contrast, LVF/low-contrast, UVF/high-contrast, LVF/high-contrast). We estimated the 95% confidence intervals of the correlation coefficient by bootstrap resampling with 10,000 repetitions. Bootstrap analysis was conducted by using the MATLAB Statistics and Machine Learning Toolbox.

#### Evaluating tissue properties of white matter tracts

We evaluated the tissue properties of white matter tracts (the optic radiation and the corticospinal tract) based on previously reported methods (Levin et al., 2010; Yeatman et al., 2012; Duan et al., 2015; Takemura et al., 2019; Minami et al., 2020). Briefly, we resampled individual streamlines which belong to white matter tracts to 100 equidistant nodes. The tissue properties were calculated at each node of each streamline using spline interpolation of the tissue properties quantified by a diffusion tensor model (FA and MD), NODDI (ODI and ICVF), and qT1. The qT1 maps were registered with the dMRI data for each subject, and the qT1 values along each node of each streamline were computed. The properties at each node were summarized by taking a weighted average of tissue property measurement (FA, MD, ODI, ICVF, and qT1) on each streamline within that node. The weight of each streamline was based on the Mahalanobis distance from the tract core, which is calculated as the mean of each streamline’s *x*, *y*, *z* coordinates at each node (Yeatman et al., 2012). We excluded the first and last 10 nodes from the tissue property of the tract core to exclude voxels close to the gray/white matter interface where the tract is likely to be heavily intersected with other fibers, such as those in the superficial U-fibers. We then averaged 80 values at different nodes along white matter tracts for each MRI parameter to obtain subject-specific tissue properties. The measurement from the dMRI data (FA, MD, ODI, and ICVF) was averaged across two runs. Finally, we averaged each MRI parameter across the left and right hemispheres.

#### Predicting C1 latency from tissue properties of white matter

##### Full model

We fitted a multiple linear regression model in which C1 peak latency was predicted by the linear weighted sum of five MRI parameters (FA, MD, ODI, ICVF, and qT1) along the optic radiation with a constant (*c*):

(1) C1 latency=wFA * FA + wMD * MD + wODI * ODI + wICVF * ICVF + wqT1 * qT1 + c.

Model fitting was performed using the MATLAB Statistics and Machine Learning Toolbox with the objective of minimizing the least squared error by selecting the best combination of weights and constant.

##### Reduced models

We evaluated the performance of three different reduced models, which used a subset of MRI parameters.

NODDI + qT1 model:

(2) $$C1\;latency=w_{\mathrm{ODI}}\;*\;ODI\;+\;w_{\mathrm{ICVF}}\;*\;ICVF\;+\;w_{\mathrm{qT1}}\;*\;qT1\;+\;c.$$

DTI + qT1 model:

(3) $$C1\;latency=w_{\mathrm{FA}}*FA+w_{\mathrm{MD}}*MD+w_{\mathrm{qT1}}*qT1+c.$$

DTI + NODDI model:

(4) $$C1\;latency=w_{\mathrm{FA}}\;*\;FA\;+\;w_{\mathrm{MD}}\;*\;MD\;+\;w_{\mathrm{ODI}}\;*\;ODI\;+\;w_{\mathrm{ICVF}}\;*\;ICVF\;+\;c.$$

##### Full + tract length model

We also estimated a tract length of the optic radiation in each subject by calculating the mean length of the streamlines belonging to the optic radiation. The tract lengths in the left and right optic radiation were averaged. We then tested a model incorporating the estimated tract length into the full model for predicting C1 peak latency:

(5) $$C1\;latency=w_{\mathrm{FA}}*FA+w_{\mathrm{MD}}*MD+w_{\mathrm{ODI}}*ODI+w_{\mathrm{ICVF}}*ICVF+w_{\mathrm{qT1}}*qT1+w_{\mathrm{length}}*length+c.$$

##### Full + V1 cortical thickness (CT) model

We finally estimated the CT of the V1 (defined by probabilistic retinotopy atlas; Wang et al., 2015) based on the FreeSurfer segmentation (see above). Next, we tested a model that incorporated the V1 CT into the full model for predicting C1 peak latency:

(6) $$C1\;latency=w_{\mathrm{FA}}\;*\;FA\;+\;w_{\mathrm{MD}}\;*\;MD\;+\;w_{\mathrm{ODI}}\;*\;ODI\;+\;w_{\mathrm{ICVF}}\;*\;ICVF\;+\;w_{\mathrm{qT1}}\;*\;qT1\;+\;w_{\mathrm{CT}}\;*\;CT\;+\;c.$$

#### Evaluation of model performance

Leave-one-out cross-validation was used to evaluate how well the models predicted C1 peak latency. Specifically, we divided the data from all subjects into 19 training datasets and one test dataset to evaluate how much each model could predict C1 latency in a subject that was not included in the model fitting phase. We iterated this procedure 20 times by changing the selection of the test dataset. We evaluated the accuracy of the model by calculating a Pearson correlation coefficient (cross-validated *R*) across the measured C1 latency and the C1 latency predicted from a multiple linear regression model.

We also evaluated the statistical significance of the model prediction using a permutation test. We randomly shuffled the association between MRI measurements and C1 peak latency across all subjects 10,000 times. After each shuffle, we performed a model prediction from MRI measurements to C1 peak latency using a multiple linear regression model and leave-one-out cross-validation to obtain a correlation coefficient (*R*). We then calculated the percentile of the original correlation coefficient with respect to the distribution of the correlations calculated over the 10,000 permutations. This percentile is reported as the *p* value, which is the likelihood of randomly acquiring the original correlation value.

For the full model, to estimate the contribution of each MRI parameter, we also performed a multiple linear regression analysis on data from 20 subjects without performing a leave-one-out cross-validation. We reported the *t* value and *p* value for each MRI parameter on the multiple linear regression of the full model to quantify the contribution of each MRI parameter with regard to latency prediction.

### Software accessibility

The code for reproducing figures and statistical analyses in this work is publicly available online via a public repository (https://github.com/htakemur/PredictingLatencyfromOR). The code was written in MATLAB and tested in MATLAB 2015a on Ubuntu 14.04 LTS.

## Results

We sought to test the extent to which the interindividual difference in C1 latency depends on the MRI-based tissue properties measurements of the optic radiation. While several studies have previously demonstrated the test-retest reliability of dMRI and qT1 measurements (Vollmar et al., 2010; Mezer et al., 2013; Chung et al., 2016), such analyses are rarely conducted on MEG measurements. Therefore, we first evaluated the variability and test-retest reliability of the MEG measurements on C1 latency across the different stimulus conditions. We then tested the extent to which MRI measurements of the optic radiation could predict interindividual variability in C1 latency under the stimulus condition with the highest test-retest reliability. We also evaluated the accuracy of C1 prediction from a non-visual white matter tract (the corticospinal tract). Finally, we assessed how much tissue measurements along the optic radiation could predict C1 latency in response to visual stimuli presented in the contralateral visual field.

### Distribution, stimulus dependence, and test-retest reliability of C1 peak latency

**Figure 2.**
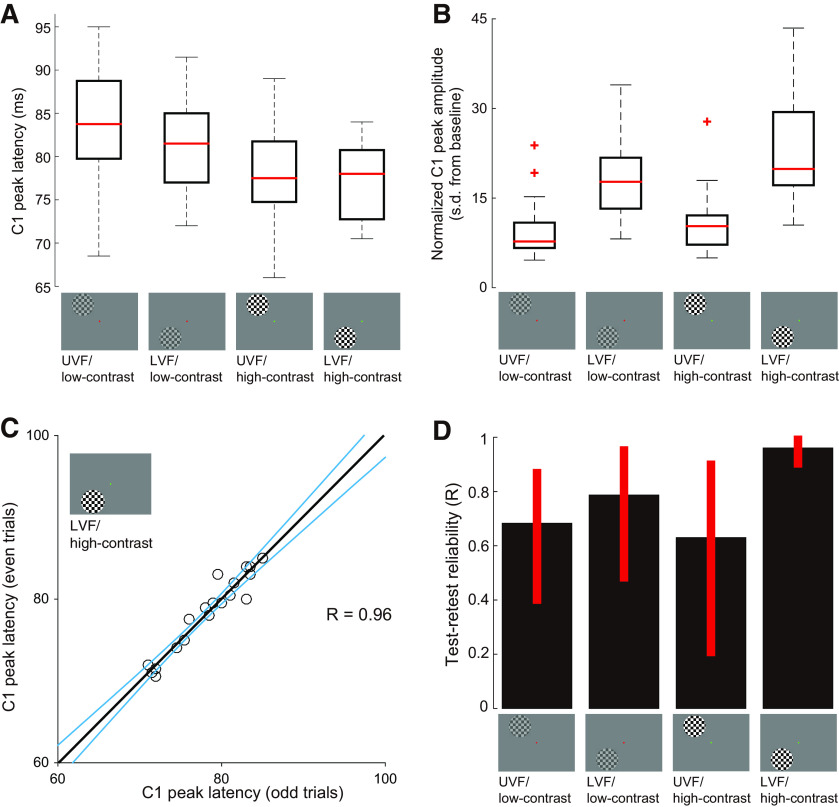
Properties of the C1 peak latency/amplitude measured by MEG. ***A***, Distribution of C1 peak latency in four stimulus conditions (UVF/low-contrast, LVF/low-contrast, UVF/high-contrast, LVF/high-contrast). Median C1 latencies in each condition are depicted as red lines. The border of the black box indicates the 25% and 75% percentiles in each condition. The error bars with a dotted line indicate the range of C1 latency for all subjects. ***B***, Distribution of the C1 peak amplitude in four stimulus conditions. The vertical axis depicts the extent to which C1 peak amplitude was deviated from mean response amplitude during the baseline period (−200 to −1 ms from the stimulus onset) with a unit of SD of the response amplitude within the baseline period. The red cross indicates outlier data (outside ± 2.7 SDs within a distribution in each condition). Other conventions are identical to those in ***A***. ***C***, Test-retest reliability in LVF/high-contrast condition. The scatter plot compares C1 peak latency in odd trials (horizontal axis) and even trials (vertical axis). Each individual dot depicts the C1 peak latency in each individual subject and the black line is a linear regression line. The blue curves indicate the 95% confidence interval of a linear regression estimated by the bootstrapping method. ***D***, Test-retest reliability under all stimulus conditions. The vertical axis depicts the correlation coefficient (*R*) of C1 peak latency between the odd and even trials. The error bar depicts the 95% confidence interval of the correlation coefficient estimated by the bootstrapping method (see Materials and Methods).

First, we evaluated the extent to which the properties of C1 depend on the stimulus conditions used in our MEG experiment (UVF or LVF; low- or high-contrast; the data in the left and right visual field stimulus presentation were averaged). Figure 2*A* shows a box plot of C1 peak latency in each stimulus condition. Under all conditions, the C1 peak latency ranged from 65 to 95 ms after the stimulus onset. The range of C1 peak latency was consistent with the range reported in a previous study that used similar checkerboard stimuli (Di Russo et al., 2002). The median C1 peak latencies across subjects were 83.8, 81.5, 77.5, and 78.0 ms for each stimulus condition (UVF/low-contrast, LVF/low-contrast, UVF/high-contrast, and LVF/high-contrast, respectively). We also found notable interindividual differences in C1 peak latency (with SDs of 7.4, 5.2, 6.2, and 4.3 ms for each stimulus condition). There was no significant correlation between C1 peak latency and subject age under all stimulus conditions (*R *=* *0.11, 0.12, −0.13, and 0.04; *p *=* *0.65, 0.62, 0.57, and 0.88, for each stimulus condition).

To evaluate how much C1 latency depends on the stimulus condition, we performed two-way ANOVA on the C1 peak latency data (with contrast and stimulus position as main effects). We found that the main effect of contrast was significant (*F*_(1,76)_ = 12.59, *p* = 0.0007), while the main effect of visual field (upper or lower) and the interaction between contrast and visual field were not (main effect of visual field, *F*_(1,76)_ = 1.30, *p* = 0.26; interaction between contrast and visual field; *F*_(1,76)_ = 0.70, *p* = 0.41), suggesting that C1 latency was significantly delayed in the low-contrast condition.

We also evaluated the amplitude of the response at C1 peak latency (Fig. 2*B*), which was normalized using the mean and SD of the amplitude during the baseline period (−200 to −1 ms from the stimulus onset). We found that the main effect of visual field was significant (*F*_(1,76)_ = 48.17, *p* < 0.0001), while the main effect of contrast was only marginally significant (*F*_(1,76)_ = 3.37, *p* = 0.07). The interaction between visual field and contrast was not statistically significant (*F*_(1,76)_ = 0.77, *p* = 0.38). This result suggests that the amplitude of the C1 response was significantly larger for the stimuli presented at the LVF as compared with those presented at the UVF. A larger C1 response for LVF stimuli is consistent with previous reports (Portin et al., 1999; Tzelepi et al., 2001; Fortune and Hood, 2003; Hagler et al., 2009; Maruyama et al., 2009). In summary, we identified C1 peak latency within the range reported in a previous study (Di Russo et al., 2002) and found that its latency and amplitude depended on the stimulus contrast and position.

Next, we sought to identify the stimulus condition providing the most reliable C1 peak latency by assessing the test-retest reliability. To this end, we separately estimated the C1 dipole in odd and even trials (108 trials for each) and identified the C1 peak latency in each condition. We observed the highest test-retest reliability of C1 latency when high-contrast stimuli were presented at the LVF (*R* = 0.96; Fig. 2*C*) as compared with low-contrast stimuli presented at the LVF (*R* = 0.79) or low/high-contrast stimuli presented at the UVF (*R* = 0.68 and 0.63 for low- and high-contrast, respectively; Fig. 2*D*). The higher test-retest reliability in the LVF conditions may be related to the higher signal-to-noise ratio under these conditions. Considering that the test-retest reliability of C1 peak latency in the LVF/high-contrast condition far exceeded that of the other conditions, we primarily used the C1 latency data in this stimulus condition for subsequent analyses using MRI data.

### Predicting C1 latency from tissue properties of the optic radiation

**Figure 3.**
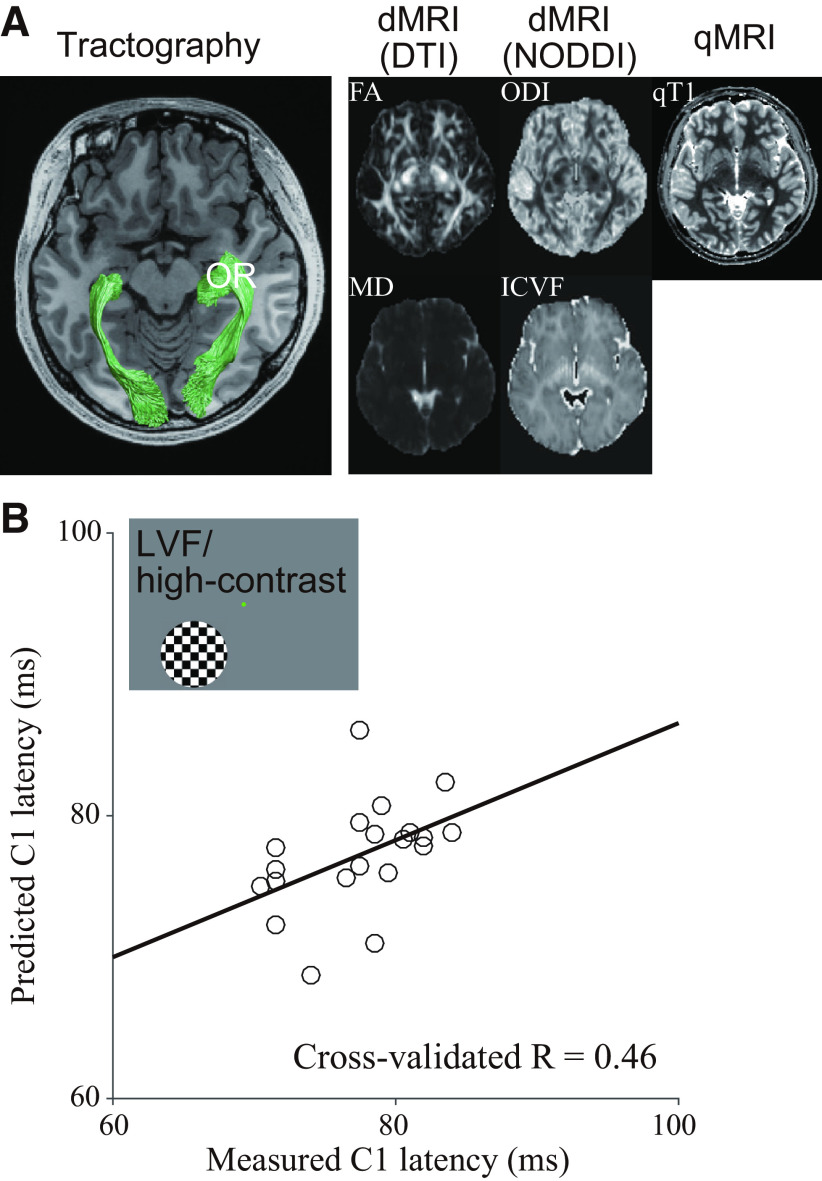
Prediction of the C1 peak latency from the tissue properties of the optic radiation (OR). ***A***, left panel, The optic radiation (green) in a representative subject (subject 9) identified using tractography in the dMRI dataset overlaid on an axial slice of a T1-weighted image. Right panel, MRI-based tissue property maps in the same subject. ***B***, Comparison between the measured C1 peak latency (horizontal axis) and the C1 peak latency predicted from the optic radiation (vertical axis) when a high-contrast stimulus was presented at the LVF. The model prediction was performed by dividing 20 subjects into 19 training datasets and one test dataset (leave-one-out cross-validation) and iterating 20 times by changing the test subject. Each datapoint indicates the measured and predicted C1 latency for an individual subject. The model showed a significant performance to predict C1 peak latency (cross-validated *R* = 0.46, *p* = 0.01). The black line depicts the linear regression between the measured and predicted C1 latency.

Using the dMRI data, we identified the optic radiation in all 20 subjects using probabilistic tractography. Figure 3*A*, left panel, represents the optic radiation identified from dMRI data in a representative subject. From the dMRI data, we estimated four tissue property measurements by using the DTI (FA and MD) and the NODDI (ODI and ICVF). We also measured qT1 by using an MRI acquisition protocol distinct from dMRI (see Materials and Methods). We then estimated five MRI parameters (FA, MD, ODI, ICVF, and qT1; Fig. 3*A*, right panel) along the optic radiation. We used these five MRI parameters, since previous works demonstrated that these parameters may be sensitive to different types of microstructural properties of brain tissues (Mezer et al., 2013; Crombe et al., 2018). We then used a multiple linear regression model to predict the interindividual variability of C1 peak latency from the five MRI-based tissue measurements along the optic radiation. We evaluated the performance of the model by leave-one-out cross-validation to test the ability of the model to predict C1 latency of the test dataset that was not used to train the model.

**Figure 4.**
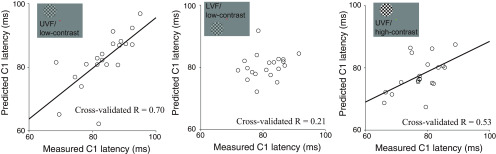
Prediction accuracy of the C1 latency in the other three stimulus conditions. Left panel, UVF/low-contrast. Middle panel, LVF/low-contrast. Right panel, UVF/high-contrast. These three conditions have a relatively lower test-retest reliability of C1 measurement (see Fig. 2*D*). Conventions are identical to those in Figure 3*B*.

Figure 3*B* depicts the comparison between the measured and predicted C1 latencies for the high-contrast stimuli presented to the LVF, which is a condition with a highest test-retest reliability (Fig. 2*D*). The model explained 22% of the variance (*R*^2^ = 0.22) in interindividual variability of C1 latency (*R* = 0.46; Fig. 3*B*). Next, we estimated the statistical significance of the model performance using a permutation test and found that its performance was statistically significant (*p* = 0.01). The result for this most reliable condition suggests that the interindividual variability of C1 peak latency can be at least partly explained by variability in tissue properties along the optic radiation. The prediction accuracy for other stimulus conditions was variable (*R* = 0.70, 0.21, and 0.53; *p* = 0.0002, 0.19, and 0.008 for UVF/low-contrast, LVF/low-contrast, and UVF/high-contrast, respectively; Fig. 4). As discussed above, given the large difference in the test-retest reliability of C1 peak latency across the stimulus conditions (Fig. 2*D*), we focused on the LVF/high-contrast condition in subsequent analyses.

In the main analysis, we used five MRI parameters to predict the variabilities in C1 latency. We sought to identify MRI parameters that contributed to predicting C1 latency in the LVF/high-contrast condition. To this end, we calculated *t* values for each MRI measurement along the optic radiation in a multiple linear regression model predicting C1 peak latency. Here, we used the data from all 20 subjects and found that all MRI parameters except for qT1 significantly contributed to the prediction of C1 peak latency in the high-contrast condition (*t* = 2.46, 2.82, 2.70, −2.41, and −0.77; *p* = 0.03, 0.01, 0.02, 0.03, and 0.45 for FA, MD, ODI, ICVF, and qT1, respectively).

**Figure 5.**
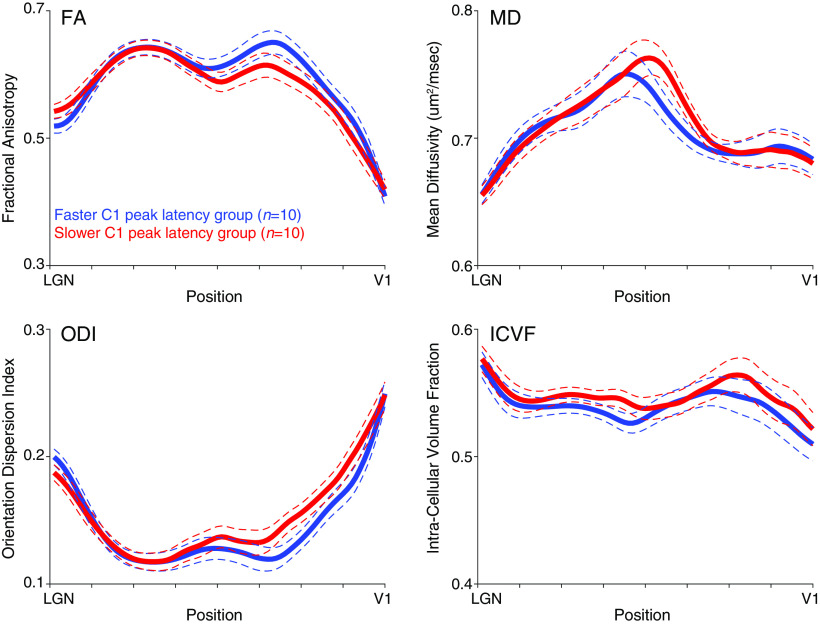
Tissue properties along the optic radiation. The four panels show the FA, MD, ODI, and ICVF measurements, respectively. The mean profiles of subjects with faster (blue, *n* = 10) and slower (red, *n* = 10) C1 peak latencies are represented as thick solid curves. The thin dotted curves represent ±1 SEM from the group mean. The horizontal axis represents the normalized position along the optic radiation (left: anterior, right: posterior).

To understand which part of the optic radiation contributed to the prediction of the C1 peak latency, we also calculated how much the spatial profile of MRI parameters depended on C1 peak latency. Figure 5 represents the spatial profile of four parameters (FA, MD, ODI, and ICVF) along the optic radiation contributing to C1 peak latency prediction in subjects with faster (*n* = 10) and slower (*n* = 10) C1 peak latencies. Group differences in each parameter were not significant (*t*_(18)_ = 1.00, −0.19, −0.96, and −0.60; *p* = 0.33, 0.85, 0.35, and 0.56 for FA, MD, ODI, and ICVF), suggesting that individual parameters may not be sufficient for C1 peak latency prediction. Meanwhile, small group differences were found mostly in the middle to posterior part of the optic radiation (closer to V1), suggesting that voxels along the straight portion of the optic radiation may contribute to C1 peak latency prediction.

**Figure 6.**
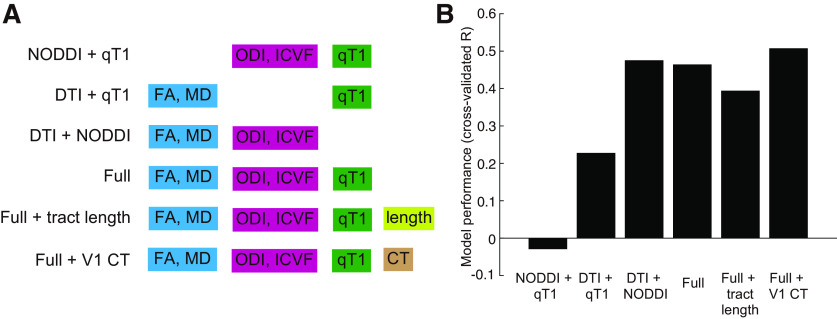
Comparison of prediction accuracy for C1 peak latency in the LVF/high-contrast condition between the models. ***A***, Schematic of the model. We compared the model tested in the main analysis (“full” model), with five variants. We tested three types of reduced models that use a subset of MRI parameters along the optic radiation (“NODDI + qT1” model; “DTI + qT1” model; “DTI + NODDI” model). We also tested a model including the streamline length of the optic radiation or CT of the V1 as an explanatory variable (“full + tract length” model; “full + V1 CT” model). ***B***, Model performance. The vertical axis shows the correlation coefficient (*R*) between the measured and predicted C1 latency in each model, which was estimated by leave-one-out cross-validation.

In the main analysis, we used five MRI parameters to measure tissue properties of the optic radiation. These parameters were estimated by different diffusion modeling methods (DTI or NODDI) or different scanning sequences (dMRI or qT1). We further tested how much variance in C1 peak latency under the LVF/high-contrast condition could be predicted by a subset of MRI parameters. We compared the performance of the full model using all five parameters with that of three reduced models using a subset of parameters (Fig. 6*A*). The performance of the model without qT1 (DTI + NODDI model; *R* = 0.48; Fig. 6*B*) was comparable with that of the full model (*R* = 0.46), consistent with insignificant contribution of qT1 shown above. The full model outperformed the other models using a subset of parameters (NODDI + qT1 model, *R* = −0.03; DTI + qT1 model, *R* = 0.23; Fig. 6*B*). These results suggest that different diffusivity parameters (FA, MD, ODI, and ICVF) may contribute to predicting C1 peak latency in a complementary way, while qT1 did not contribute.

Finally, we compared the performance of the model when including either the tract length (mean optic radiation streamline length) or the CT of the V1 as an explanatory variable in addition to the MRI parameters (full + tract length model, full + V1 CT model; Fig. 6*A*). The model incorporating streamline length had modest performance for predicting C1 peak latency (*R* = 0.39) but did not outperform the full model used in the main analysis (Fig. 6*B*). The model incorporating the CT of the V1 slightly outperformed the full model (*R* = 0.51). Therefore, while we did not find evidence that information on streamline length improves the prediction accuracy for C1 peak latency, there remains a possibility that some structural properties of the gray matter (V1) may provide further information relevant to predicting C1 latency.

### Tissue properties along the corticospinal tract did not predict variability in C1 latency

**Figure 7.**
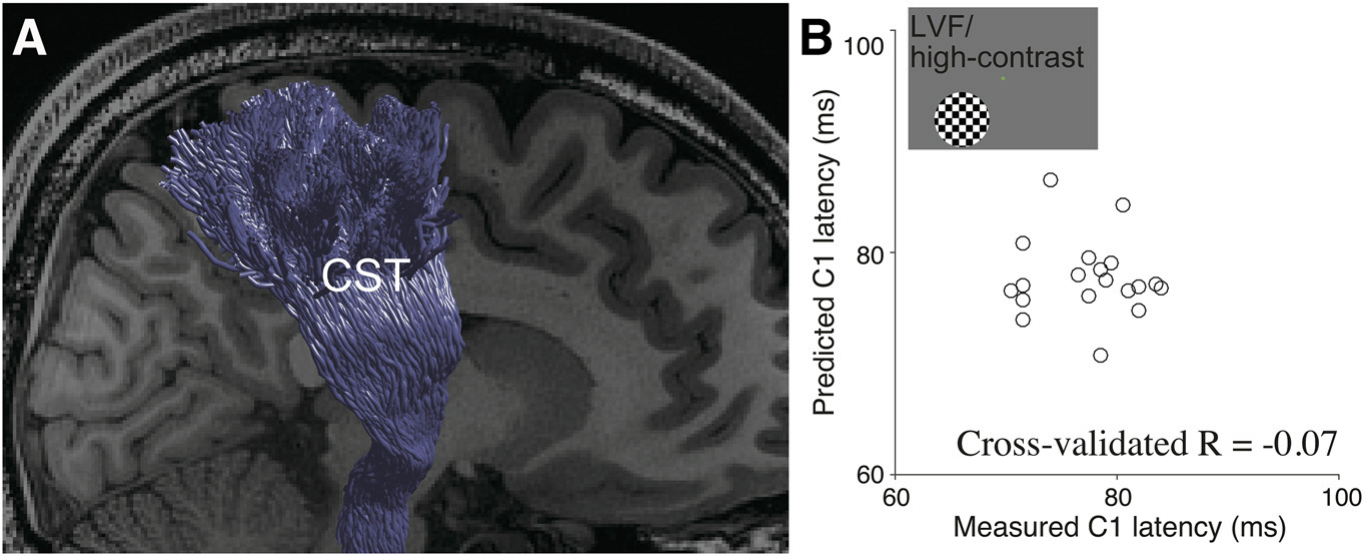
Prediction of C1 peak latency from the corticospinal tract (CST). ***A***, The CST in a representative subject (subject 9) identified using tractography in the dMRI dataset overlaid on a sagittal slice of a T1-weighted image. ***B***, Comparisons between the measured C1 peak latency (horizontal axis) and the C1 peak latency predicted from the CST (vertical axis) when a high-contrast stimulus was presented at the LVF. The model did not show a significant prediction of C1 peak latency (cross-validated *R* = −0.07, *p *=* *0.60).

We then evaluated how well the model using a non-visual tract (corticospinal tract; Fig. 7*A*) could predict C1 peak latency to clarify the extent to which prediction accuracy observed in the optic radiation is generalizable to other white matter tracts. We used an identical number of MRI parameters (FA, MD, ODI, ICVF and qT1) to predict C1 peak latency in the condition with the highest test-retest reliability (LVF/high-contrast) and evaluated the accuracy of the model using the identical leave-one-out cross-validation procedure.

The corticospinal tract model did not significantly predict interindividual variability of C1 peak latency in the LVF/high-contrast condition (*R* = −0.07, *p* = 0.60; Fig. 7*B*). We also confirmed that this model did not significantly predict C1 peak latency under any other stimulus conditions (*R* = −0.11, 0.02, and −0.34; *p* = 0.67, 0.47, and 0.92 for UVF/low-contrast, LVF/low-contrast, and UVF/high-contrast). These results suggest that the prediction accuracy observed in the optic radiation cannot be generalized to a non-visual white matter tract.

### Do tissue properties of the optic radiation predict C1 latency to stimuli presented in the contralateral visual field?

The human V1 responds predominantly to visual stimuli in the contralateral visual field. The cortical source of C1 also appears in the hemisphere contralateral to the visual field position of the presented stimuli (Di Russo et al., 2002). Therefore, we hypothesized that if we subdivided the optic radiation data into the left and right hemispheres, the tissue measurements along the optic radiation may predict the C1 peak latency to visual stimuli presented in the contralateral visual field.

**Figure 8.**
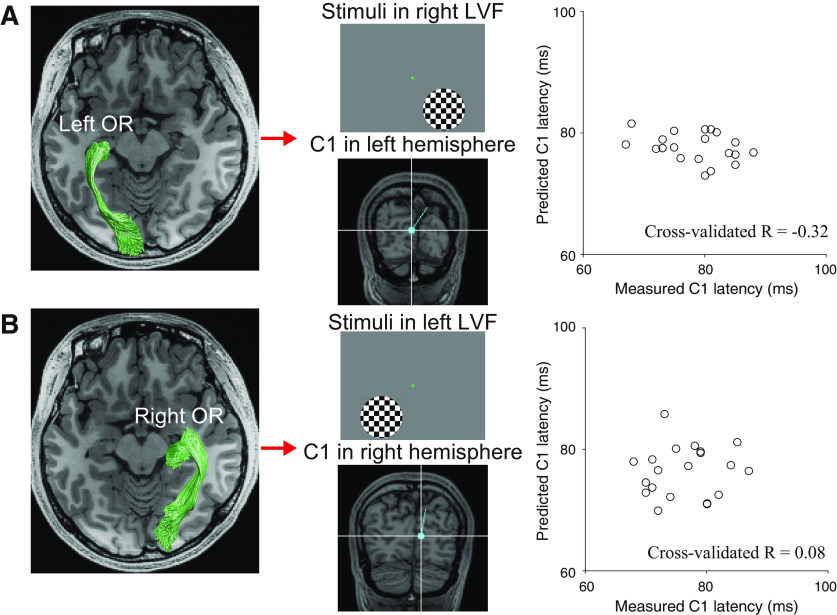
The optic radiation (OR) tissue properties from a single hemisphere did not predict C1 latency in response to high-contrast stimuli presented in the contralateral LVF. ***A***, Prediction of C1 peak latency evoked by high-contrast stimuli in the right LVF from the left optic radiation (left panel). The dipole at a time around the C1 peak latency was localized to the calcarine sulcus in the left hemisphere (middle panel, subject 9). The prediction accuracy did not reach a statistically significant level (right panel, *R* = −0.32, *p *=* *0.92). ***B***, Prediction of C1 peak latency evoked by high-contrast stimuli in the left LVF from the right optic radiation (left panel). The dipole at a time around the C1 peak latency was localized to the calcarine sulcus in the right hemisphere (middle panel). The prediction accuracy did not reach a statistically significant level (right panel, *R *=* *0.08, *p *=* *0.36) in this case as well.

As a result, the properties of the optic radiation in the left and right hemispheres cannot predict the C1 peak latency in the contralateral LVF (high-contrast) stimulation [C1 latency in the right LVF, *R* = −0.32, *p* = 0.92 (Fig. 8*A*); C1 latency in the left LVF, *R* = 0.08, *p* = 0.36 (Fig. 8*B*)]. A lack of hemispheric specificity poses a challenge when interpreting the significant prediction accuracy for averaged data and suggests that there are still challenges remaining in terms of robust prediction of the neural response latency from MRI-based tissue property measurements in white matter pathways.

**Figure 9.**
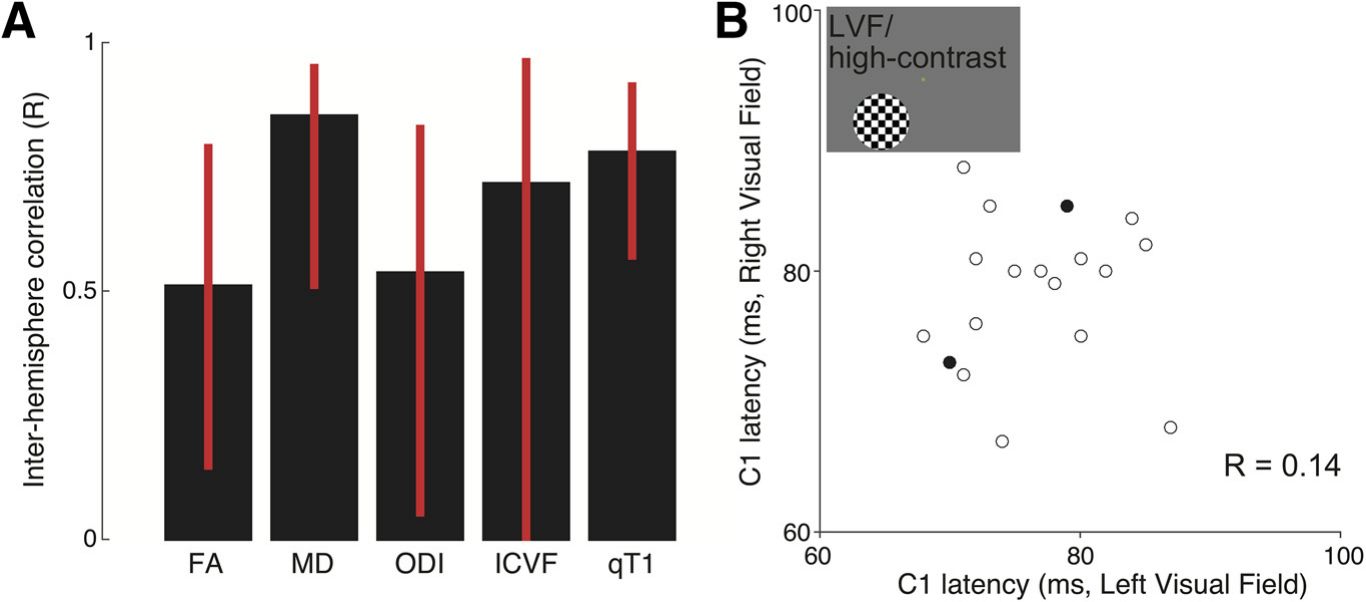
Correlation of measurements between hemispheres or visual fields. ***A***, Correlation coefficient (*R*) of MRI measurements along the optic radiation between the left and right hemispheres. The error bar depicts the 95% confidence interval estimated by the bootstrapping method. ***B***, Correlation coefficient (R) of C1 latency between the left and right visual fields (LVF/high-contrast stimulus). The open circles indicate data points from a single subject, and the filled circle shows the datapoints that overlapped between two subjects.

To clarify the reason for which there was such a large difference in prediction accuracy between the averaged and single hemisphere data, we assessed the interhemispheric correlation of MRI measurements and C1 peak latency (Fig. 9). The MRI measurements along the optic radiation correlated across the hemispheres, while a degree of correlation varies across metrics (*R* = 0.51, 0.85, 0.54, 0.72 and 0.78 for FA, MD, ODI, ICVF, and qT1, respectively; Fig. 9*A*). In contrast, the C1 peak latency under the LVF/high-contrast condition did not correlate between stimulation to the left and right visual field (*R* = 0.14; Fig. 9*B*). We noted that the test-retest reliability of the C1 peak latency in each visual field was considerably high (right LVF/high-contrast, *R* = 0.91; left LVF/high-contrast, *R* = 0.94) such that the hemispheric differences in C1 peak latency are reproducible, rather than a product of unstable MEG measurements. These findings suggest that the lack of hemispheric specificity might be due to the fact that MRI measurements are correlated across hemispheres while MEG measurements are not. This may be a simple consequence of the fact that MRI measurements have insufficient sensitivity to be able to identify interhemispheric latency differences, since some estimates of MRI measurements in a single hemisphere may be noisy given a relatively lower interhemisphere correlations in FA and ODI or a larger confidence interval of interhemisphere correlation in ICVF (Fig. 9*A*). Alternatively, it is also plausible that an anatomical or physiological factor other than the tissue properties of the optic radiation may be eliciting a hemispheric difference in C1 latency.

## Discussion

The aim of this study was to determine the ability of MRI-based tissue measurements along the human optic radiation to predict interindividual variability in C1 peak latency, which is the earliest component of visually evoked responses. Analysis of the optic radiation data averaged across the hemispheres predicted 22% of variance in C1 peak latency for high-contrast stimuli presented in the LVF, for which we obtained the highest test-retest reliability of C1 peak latency. Analysis of the corticospinal tract revealed that the prediction accuracy observed in the optic radiation may not be generalizable to non-visual white matter tracts. The optic radiation measurements along the left/right hemisphere failed to predict C1 peak latency to visual stimuli presented in the contralateral visual field. In summary, we found evidence that interindividual variability in C1 peak latency can be explained in part by the tissue properties of the optic radiation under specific stimulus conditions. Below, we discuss other possible factors which may further explain interindividual variability in C1 peak latency, the relationship between this study and previous literature, and current limitations in non-invasive MRI and MEG methods.

### What factors may further explain interindividual variability in C1 peak latency?

In this study, we found evidence to suggest that the tissue properties of the optic radiation in part explain interindividual variability in C1 latency. However, there are several other factors that may be relevant for C1 peak latency.

#### Latency difference in the retina

Another factor that could account for the interindividual variability in C1 peak latency is interindividual variability in response latency in retinal cells. In humans, this has been widely assessed using the electroretinogram (ERG; McCulloch et al., 2015). However, interindividual variability of ERG peak latency is reported to be very small in healthy subjects (e.g., the SD in healthy subjects was 1–2 ms in a previous study; Gauvin et al., 2014). Therefore, it is unlikely that variability in latency of retinal cells would explain a large part of variance in interindividual variability of C1 peak latency, which is on the order of 10–20 ms (Figs. 2*A*, 3*B*).

#### Pupil size and retinal illumination

Previous studies demonstrated a significant relationship between pupil size and latency of visually evoked responses by artificially varying the pupil size of human subjects (Hawkes and Stow, 1981; Martins et al., 2003). Another line of study demonstrated that latency of visually evoked response was delayed by decreasing retinal illumination (Froehlich and Kaufman, 1991). Given that previous studies demonstrated interindividual difference in pupil size (Higuchi et al., 2008; Aminihajibashi et al., 2019), since we did not control for retinal illumination, we cannot exclude the possibility that the variability in these factors during the MEG experiment may have affected the measurements of C1 peak latencies. Therefore, incorporating these factors may improve the precision of the C1 latency prediction from MRI measurements on the optic radiation.

#### Optic nerve and optic tract

In this study, we could not incorporate the tissue properties of the earlier visual white matter tract, i.e., the optic nerve and optic tract, into the model used to predict variance in C1 peak latency because of the greater difficulties involved in obtaining reliable measurements from these tracts as compared with the optic radiation. The optic nerve is particularly difficult to measure using standard dMRI acquisitions because it is prone to susceptibility-induced distortions and signal dropout. The optic tract is also prone to measurement difficulties because of a relatively small signal-to-noise ratio and small volume, which may cause partial volume effects with cerebrospinal fluid. It may be challenging to perform this type of analysis using a higher-order model like NODDI to assess tissue properties in the optic tract. Advanced measurement methods, such as readout-segmented EPI, hold promise in terms of improving the quality of dMRI measurements in these tracts (Porter and Heidemann, 2009; Frost et al., 2015; Kida et al., 2016) and providing more information to predict C1 peak latency in future investigations.

#### Tract length

Variability in the tract length could also explain the interindividual difference in C1 peak latency. To explore this hypothesis, we included tract length (mean length across all optic radiation streamlines in each subject) as an explanatory variable in the model but did not find any improvement in prediction accuracy (see Results). A potential limitation of this approach is that streamlines only approximate the trajectory of fiber bundles and are not true axons, so may not fully capture interindividual variability in length of the optic radiation fibers. Indeed, fibers in the optic radiation may change their position along the tract (Nelson and LeVay, 1985), and it is not fully clear whether or not streamline lengths are useful for approximation of fiber length. Better understanding of the significance of fiber length may require additional assessment using anatomical methods and advanced modeling.

#### Difference in latency derived from processing of neural information in gray matter

Peak latency measured using MEG or EEG may reflect build-up process of local field potential but does not directly reflect the response latency of spiking activity in single-neuron electrophysiology. Such a response profile may involve multiple physiological factors, such as summation of local synaptic activity across neurons (Monosov et al., 2008) or the degree of synchrony between neurons in the gray matter (Hermes et al., 2017). In fact, incorporating the CT of V1 into the model provided a modest improvement in the accuracy of the latency prediction (Fig. 6), although the neurobiological interpretation of MRI-based estimates on CT remains an area of active investigation (la Fougère et al., 2011; Wagstyl et al., 2020). An improved understanding of the relationship between anatomy and physiology is required to understand what type of anatomical features are useful for characterizing interindividual latency variability derived from neural information processing in the gray matter.

#### Cortical feedback

A number of anatomical, electrophysiological, and neuroimaging studies have demonstrated the existence of feedback signal from the extrastriate cortex to the V1 (Rockland and Virga, 1989; Lamme et al., 1998; Girard et al., 2001; Muckli et al., 2015; Rockland, 2020). A study on non-human primates revealed that feedback signals from extrastriate cortex affect visually-evoked responses of V1 neurons at a very early phase (i.e., 10 ms after response onset; Hupé et al., 2001). Therefore, it is likely that not only feedforward but also feedback signals from extrastriate areas affect the C1, limiting the accuracy of the C1 peak latency prediction solely based on the structural properties of the optic radiation.

### Related studies

Previous studies have demonstrated delayed visually evoked responses in patients with demyelinating diseases, such as multiple sclerosis (Halliday et al., 1972; Thurtell et al., 2009). More recent studies have demonstrated a correlation between diffusivity measurements along the early visual white matter pathway and the latency of visually evoked responses in multiple sclerosis patients (Alshowaeir et al., 2014; Takemura et al., 2017). Price et al. (2017) also demonstrated that an age-related delay in the visually evoked response can be predicted from diffusivity measurement on the optic radiation. These studies suggest that MRI-based white matter measurements could provide useful information for prediction of variability in visually evoked responses, if such interindividual variability in latency was disease-related or age-dependent. The present results suggest that such predictive power may be at least partly generalizable to relatively small interindividual differences between healthy subjects.

Horowitz et al. (2015a) tested the relationship between dMRI-based measurements (AxCaliber or FA) of white matter properties and conduction velocities measured by EEG. Although the interpretation of their findings has remained controversial (Horowitz et al., 2015b; Innocenti et al., 2015), these authors successfully demonstrated a correlation between white matter measurements and latency measurements in healthy subjects (Horowitz et al., 2015a). One notable difference from our study is that they investigated a correlation between white matter measurements in the corpus callosum and interhemispheric delay of visual or tactile evoked responses measured by EEG. Since the corpus callosum has a relatively uniform fiber orientation within voxels, and a large number of histological measurements have been performed, it may be relatively easy to make an inference about the underlying microstructure from MRI-based measurements (Barazany et al., 2009; Alexander et al., 2010; Huang et al., 2015; Stikov et al., 2015; Berman et al., 2018, 2019; Drakesmith et al., 2019; Veraart et al., 2020). In contrast, fibers in the optic radiation change their orientation and position within a tract before reaching the terminal (Nelson and LeVay, 1985) and also cross with other neighboring pathways (Chamberland et al., 2017). We speculate that the lack of hemisphere-specific correlation in this study may be partly due to the fact that identifying the microstructural properties using MRI measurements in the optic radiation is more difficult than in the corpus callosum.

### Current challenges in MRI and MEG measurements

This study did not provide evidence of generalization across all different stimulus conditions (Fig. 4) and of hemispheric specificity (Fig. 8). These results may reflect some limitations in this study. One notable limitation is the relatively smaller sample size (*N* = 20) used to elucidate interindividual variability, which may have limited the statistical power of the study. However, in addition to a limitation in statistical power, there are several existing challenges to current non-invasive MRI and MEG methods for establishing associations between structural measurements in white matter pathways and measurements of neural response latency in humans.

In addition to the issues related to the signal qualities in the optic nerve and optic tract, as discussed above, dMRI measurements have limited spatial and angular resolution. Improved measurement methods may improve our ability to assess the properties of tissue in the visual white matter pathways without influence of a partial volume effect with other neighboring pathways or cerebrospinal fluid. Furthermore, improved dMRI data acquisition method may improve the accuracy of tractography on the optic radiation (Chamberland et al., 2018). There are ongoing efforts to improve the signal quality and resolution of dMRI (Setsompop et al., 2018; Roebroeck et al., 2019) and to develop post-processing methods on improving dMRI data resolution (Alexander et al., 2017).

dMRI-based tractography is an excellent approach to identifying trajectories of major white matter pathways like the optic radiation (Raz and Levin, 2014; Rokem et al., 2017). However, in terms of current knowledge, it is not fully clear how much variance in MRI measurements along optic radiation voxels can be explained by properties of feedforward pathways from the LGN to V1, because there may be other pathways that partly pass through the same white matter regions. Anatomical studies in non-human primates have reported the existence of feedback connections from V1 to the LGN (Ichida and Casagrande, 2002; Angelucci and Sainsbury, 2006). Although Heinrich Sachs, a classical neuroanatomist, proposed that feedforward and feedback pathways may pass through a different white matter region (Sachs, 1892), the spatial organization of these pathways along the optic radiation in humans is not well understood. Moreover, other studies reported the existence and importance of pathways connecting the pulvinar and visual cortex (Kaas and Lyon, 2007; Bridge et al., 2016; Baldwin et al., 2017). It may be that the pulvino-cortical pathways in humans pass partly through the common voxel as LGN-V1 pathways at the resolution of dMRI. It is likely that contamination between the feedforward LGN-V1 pathway and other pathways within the same voxel poses a challenge for predicting V1 latency from a structural MRI dataset.

MEG measurements also pose challenges in terms of comparison with dMRI and qT1 data. For example, if we could obtain reliable peak latency measurements from the LGN, we could calculate the conduction velocity from the LGN to V1 for comparison with the tissue properties of the optic radiation. While a recent study has reported that the early peak of a visually evoked response can be localized to the LGN (Yoshida et al., 2017), it is difficult to obtain such a response in a consistent manner across all subjects. We also note that, while most studies have reported that C1 primarily originates from V1, we could not fully exclude the influence of signals from neighboring areas (such as V2 and V3), due to the limitations inherent to the precise estimation of source localizations.

We may also need to improve biophysical models to better understand the relationship between MRI measurements and underlying white matter microstructure. While microstructural modeling of MRI data for the corpus callosum has been successful (Horowitz et al., 2015a; Stikov et al., 2015; Assaf et al., 2008; Berman et al., 2018, 2019; Drakesmith et al., 2019; Veraart et al., 2020), generalization from the corpus callosum to the optic radiation may require additional work and validation. Another recent study also found that axonal conduction velocity depends not only on the myelin g-ratio (the ratio between the inner and outer diameters of the myelin sheath) but also on myelin internode length highlighting a need to include additional microstructural information to further understand the conduction velocity (Etxeberria et al., 2016).

Finally, although peak latency is fairly reproducible and widely mentioned in the literature, the extent to which peak latency could represent the neuronal response latency in the visual system is debatable. For example, a limitation of peak latency is that it does not distinguish between a signal that starts early and rises slowly and a signal that starts late and rises rapidly (Norcia et al., 2015). Modeling of the relationship between the MEG signal and the underlying neuronal response properties will be essential to reduce the limitations of MEG measurements of neuronal latency in future investigations.

In conclusion, we found that individual differences in latency of the early visually evoked response in humans can be partly explained by the differences in tissue along the optic radiation. Although the model using tissue properties of the optic radiation explained >20% of variance in C1 peak latency, other factors may need to be incorporated into the model to improve our understanding of the structural-functional relationship in the early visually evoked response in humans.

## Synthesis

Reviewing Editor: Bradley Postle, University of Wisconsin Decisions are customarily a result of the Reviewing Editor and the peer reviewers coming together and discussing their recommendations until a consensus is reached. When revisions are invited, a fact-based synthesis statement explaining their decision and outlining what is needed to prepare a revision will be listed below. The following reviewer(s) agreed to reveal their identity: Janine Mendola, Mark Greenlee.

Here is the full text of the two reviews:

R1

This manuscript examines possible structural MRI correlates of the latency of the human visual evoked potential (VEP) response. The authors focus on the white matter of the optic radiations. Quite a bit of effort is devoted to exploring the effects of visual stimulus strength and location on the VEP itself, and in determining which combination produces the most reliable estimate of VEP latency. This is reasonable. It might be pointed out however that estimates of the reliability of the MR measures were not available. Nevertheless, the methods seem competent and the multiple regression based statistics adequate. Ultimately, the amount of variance explained was significant but modest (20%), and was shown not to generalize to control measures from another white matter tract. However, the lack of correlation for each individual hemisphere was confusing. The authors did admit this, and devoted some discussion to the issue, but no definitive answers. One potentially important source of error that should be discussed is the source localization of the MEG. Overall, I found the manuscript to be conceptually straight forward, but not really theory or model driven, with moderate novelty. Perhaps the modest impact could be improved with revisions. Specific comments follow.

Introduction

The authors provide a good scientific background, but I was left wondering about any applied or clinical reasons why is it important to understand variability in VEP latency, across multiple conditions ?

Methods & Results

The age range and standard deviation should be stated, not just mean.

It would be good to provide some more rationale for choosing the single equivalent dipole method to analyze the MEG. Some more justification is needed. I noted that that MRI data was acquired from all the subjects, so it might have been possible to guide source localization more accurately via a minimum-norm technique with reconstructed cortical surfaces. Also, please clarify if a single dipole was chosen for each hemisphere separately, and how that might impact the lack of model prediction for the contralateral visual field (in the Results section)

Discussion

The possibility of cortical feedback effects (even in primary visual cortex) should not be ignored. These effects may be substantial (especially given that within-subject variability was as high as between-subject).

As stated above, another source of error that could be listed is source localization estimates.

R2

The study explores the correlation between diffusion properties of the visual white matter (optic radiation) and the MEG-measured latency of the early (C1) evoked potential in 20 healthy participants. The work appears to have been carefully conducted and the data acquistion and analysis appears to be state of the art. The results point to a modest, but statistically significant, correlation between linearly combined white-matter diffusion properties (FA, MD, ODI and ICVF) and C1 latency for high contrast checkerboard stimuli presented in the lower visual field. Other stimulus conditions did not yield significant correlations. The coefficient of determination (R-squared) suggested that around 20% of the C1-latency variance could be accounted for by the variations in the diffusion properties of the optic radiation. Control comparisons point to the specificity of this effect.

Overall the study makes a contribution to our knowledge of how DWI can be used to explore variability of evoked activity in the human visual pathway. There is need for some clarifications (see below).

1) As explained on pp. 11-12, the authors carefully segmented the 50,000 streamlines to estimate diffusion values along the optic radiation. However, the results of this analysis are no longer mentioned. It would be interesting for the reader to have a figure depicting how FA/MD/ICVFand ODI vary ALONG the optic radiation. One could divide the 20 participants into 2 groups based on a C1-latency median split to determine where along the optic radiation significant differences occur in diffusion properties.

2) The qT1 values appear to contribute little to estimations of C1 latency (see model comparisons in Fig. 6 and the respective text). However, the authors did not estimate the cortical thickness (CT) in the respective projection zones of primary visual cortex. Individual differences in CT could be indicative of local neural processing in that part of visual cortex and could thus contribute to explained variance of the C1 latency. It should be easy to add this information into the multiple regression models depicted in Fig. 6 and elsewhere.

3) The red error bars in Fig. 9 suggest that correlation between left and right visual cortices is high for MD and qT1 and the variability of this coefficient to low, whereas the opposite is true for FA, ODI and ICVF. The latter suggests the estimates in the two hemispheres are noisy, which would limit their predictive power. The authors should comment on these findings.

4) In the Discussion section the authors consider other factors that could contribute to the variability of their results. They should also consider differences in pupil size and retinal illumination, which will affect the C1 latency and these factors will vary across subjects.

Minor point

5) Please add a trace (e.g. in light grey) for the evoked C1 component in Fig. 1B right panel. This would give the reader more information about the effect of stimulus contrast.
